# Correlation between psychological stress levels and the severity of overactive bladder symptoms

**DOI:** 10.1186/s12894-015-0009-6

**Published:** 2015-03-08

**Authors:** Henry Lai, Vivien Gardner, Joel Vetter, Gerald L Andriole

**Affiliations:** Division of Urologic Surgery, Department of Surgery, Washington University School of Medicine, 4960 Children’s Place, Campus Box 8242, St Louis, MO 63110 USA; Department of Anesthesiology, Washington University School of Medicine, 4960 Children’s Place, Campus Box 8242, St Louis, MO 63110 USA

**Keywords:** Psychological stress, Overactive bladder, Urgency incontinence, Urinary urgency, Interstitial cystitis

## Abstract

**Background:**

The relationship between psychological stress and interstitial cystitis/bladder pain syndrome (IC/BPS) has been well described. Even though there is some overlapping of symptoms between overactive bladder (OAB) and IC/BPS, there have been very few studies that specifically investigated the relationship between psychological stress and urinary symptoms in OAB patients who do not have pelvic pain. Here we examined the relationship between psychological stress levels and the severity of overactive bladder (OAB) symptoms.

**Methods:**

Patients diagnosed with OAB (n=51), IC/BPS (n=27), and age-matched healthy controls (n=30) participated in a case control study that inquired about their psychological stress levels using the perceived stress scale (PSS). PSS reported by the three patient groups were compared. Among OAB patients, their responses on the PSS was correlated to OAB symptoms using the following questionnaires: 1) international consultation on incontinence – urinary incontinence (ICIQ-UI), 2) international consultation on incontinence – overactive bladder (ICIQ-OAB), 3) OAB-q short form, 4) urogenital distress inventory (UDI-6), 5) incontinence impact questionnaire (IIQ-7), 6) urgency severity scale (USS), 7) numeric rating scales of urgency symptom, and 8) frequency symptom. Spearman’s correlation tests were performed to examine the relationship between psychological stress levels and the severity of OAB symptoms.

**Results:**

OAB patients reported psychological stress levels that were as high as IC/BPS patients (median 17.0 versus 18.0, p=0.818, Wilcoxon sum rank test), and significantly higher than healthy controls (17.0, versus 7.5, p=0.001). Among OAB patients, there was a positive correlation between perceived stress levels and urinary incontinence symptoms (ICIQ-UI, Spearman’s correlation coefficient=0.39, p=0.007), and impacts on quality of life (UDI-6, IIQ-7, OAB-q quality of life subscale; Spearman’s correlation coefficient=0.32, 0.31, 0.39, and p=0.028, 0.005, 0.029, respectively). No significant correlation was observed between perceived stress levels and urgency or frequency symptoms (ICIQ-OAB, USS, numeric ratings of urgency and frequency).

**Conclusions:**

OAB patients reported psychological stress levels that were as high as IC/BPS patients, and significantly higher than healthy controls. There was a positive correlation between perceived stress levels and urinary incontinence symptoms, and its impacts on quality of life among OAB patients.

## Background

The relationship between psychological stress and interstitial cystitis/bladder pain syndrome (IC/BPS) has been well described. Many IC/BPS patients reported that stress exacerbates their bladder symptoms, including urgency [1,2]. There is a positive correlation between psychological stress levels and the severity of urgency and bladder pain symptoms in IC/BPS [3]. This positive correlation becomes progressively stronger in patients with more severe IC/BPS symptoms [3]. Even though there is some overlapping of symptoms between OAB (overactive bladder) and IC/BPS [4], surprisingly there have been very few studies that specifically investigated the relationship between psychological stress and urinary symptoms in OAB patients who do not have pelvic pain.

Knight et al. compared the recent life stress measures between “OAB dry” patients (without urgency incontinence) and age-matched controls, and found no differences in life stress scores between the two groups [5]. Unfortunately the authors did not recruit “OAB wet” patients with incontinence for comparison. Zhang et al. conducted an epidemiological study to compare the occupational stress levels between female adult Chinese nurses who had OAB-like symptoms versus nurses who did not have OAB-like symptoms [6]. The authors concluded that nurses with OAB-like symptoms had higher occupational stressors and higher psychological strain than nurses without OAB. There are several limitations of that study: (1) the study population were nurses but not a clinical population of OAB patients seeking treatments; (2) the nurses did not have an evaluation or a diagnosis of OAB by a clinician; (3) the OAB-like symptoms reported on the questionnaires may not be bothersome; and (4) the authors used a validated questionnaire that specifically examined occupational stressors but not everyday psychological stressors. Thus, it is difficult to infer the results of the Chinese nurse study to the OAB patients [6].

The objectives of this study were to: (1) compare perceived stress levels in patients with OAB to IC/BPS and controls, and (2) examine the correlation between the level of psychological stress and the severity of OAB symptoms, and its impact on quality of life in patients with OAB.

## Methods

### Population

Between October 2012 and July 2014, patients diagnosed with OAB or IC/BPS, and healthy controls (age matched to OAB) were recruited into this questionnaire-based study that inquired about their perceived stress levels and OAB symptoms. For OAB, patients must have urinary urgency, with or without urgency incontinence, usually with frequency and nocturia, in the absence of infection or other identifiable causes, in accordance with the 2002 ICS definition of OAB [7]. For IC/BPS, patients must have an unpleasant sensation (pain, pressure, discomfort) perceived to be related to the bladder, associated with lower urinary tract symptoms of more than 6 weeks duration, in the absence of infection or other identifiable causes, consistent with the 2011 AUA IC/BPS Guideline [8]. The clinical assessment followed the published AUA guidelines [8,9]. The majority of OAB patients were undergoing treatments as recommended by the AUA OAB Guideline [9]. OAB patients with concomitant stress incontinence (or mixed incontinence) were also eligible if they reported predominant urgency incontinence by history. Patients with a history of prostate surgery, urinary incontinence surgery, urethral stricture, neurogenic bladder, urinary retention, pelvic radiation, tuberculosis cystitis, cyclophosphamide cystitis, genitourinary cancer, urinary stones, or a documented positive urine culture in the past 6 weeks were excluded. Patients with a positive culture or a post-void residual ≥150 mL (bladder scanner) on the day of visit were excluded.

Age-matched healthy controls were recruited by local advertisement and research database. Controls had no prior diagnosis of OAB or IC/BPS, no significant lower urinary tract symptoms (AUA symptom index < 7), no significant bladder or pelvic pain, and no evidence of infection. Controls were age-matched to the OAB group. All participants signed an informed consent. The Washington University School of Medicine Institutional Review Board approved this study.

### Assessment

Perceived stress was measured using the validated 10-item perceived stress scale (PSS) at the time of enrollment to the study [10]. The PSS measures the degree to which situations are perceived as being unpredictable, uncontrollable and overwhelming during the previous month. High scores indicate higher perceived stress.

Participants also completed the following validated questionnaires at the same time to assess their self-reported OAB symptoms and their impact: 1) international consultation on incontinence – urinary incontinence short form (ICIQ-UI) [11], 2) international consultation on incontinence – overactive bladder (ICIQ-OAB) [12], 3) OAB-q short form [13], 4) urogenital distress inventory short form (UDI-6) [14], 5) incontinence impact questionnaire short form (IIQ-7) [14], 6) urgency severity scale (USS) to assess the severity of urgency symptoms [15], 7) numeric rating scale (0 to 10) of the severity of urgency symptom, and 8) numeric rating scale (0 to 10) of the severity of frequency symptom. ICIQ-UI is a 4-item questionnaire that assesses the frequency, amount and interference of urinary incontinence. ICIQ-OAB is a 4-item questionnaire that inquires about daytime frequency, nighttime frequency, urgency, and urgency incontinence. OAB-q contains two sub-scales (symptom bother, quality of life) that assess symptom bother and health-related quality of life of both continent and incontinent OAB patients. UDI-6 and IIQ-7 measures urinary distress and incontinence impact, respectively. USS is a 4-point rating of the degree of urgency sensation (none, mild, moderate, severe). Additional psychosocial factors including childhood traumatic events, anxiety, and depression were assessed using the childhood traumatic event scale and hospital anxiety and depression scale (HADS), respectively [16,17]. Obesity data were not recorded.

### Statistical analyses

Wilcoxon rank sum tests were used to compare perceived stress results from OAB, IC/BPS, and controls. Multivariate linear models were used to compare PSS between the three groups adjusting for age and sex. There were 79 omitted answers out of 5076 possible for the variables of interest (1.6%). Missing data were random and were ignored for the comparisons. p < 0.05 was considered significant.

Spearman’s correlation analysis was performed to examine the relationship between perceived stress levels and self-reported OAB symptoms. p-values were calculated for the correlation using two-tailed tests using a *t* approximation testing the null hypothesis of no correlation. p < 0.05 was considered significant. All statistical analysis was completed using the open source statistical package R v2.15.1 [18].

## Results

### Demographics

51 OAB patients, 27 IC/BPS patients, and 30 healthy controls (age matched to OAB) participated in this case control study. The mean age (± SD) of the OAB, IC/BPS and control groups was 53.7 ± 11.9, 44.8 ± 16.6, 54.2 ± 12.3, respectively. There was no age or sex difference between OAB patients and healthy controls (p = 0.984 and 0.14 respectively). OAB patients were significantly older than IC/BPS patients (p = 0.013). OAB patients were also less likely to be females compared to IC/BPS (73% versus 100%, p = 00027, Chi-square test). Their demographics and clinical characteristics were presented in Table 1.

**Table 1:** **Demographics and clinical characteristics**

	**OAB**	**IC/BPS**	**Controls**
No. of participants	51	27	30
Age (mean ± SD)	53.4 ± 11.9	44.8 ± 16.6	54.2 ± 12.3
Sex (% females)	73%	100%	57%
Age of diagnosis of OAB or IC/BPS (mean ± SD)	47.5 ± 15.2	38.8 ± 10.3	NA
% with OAB or IC/BPS symptoms less than one year? (early onset cases)	24%	22%	NA
***Comorbidities:***			
Hypertension	37%	30%	33%
Diabetes	8%	0%	3%
Stroke, TIA	8%	0%	7%
Angina, MI	0%	0%	0%
Depression	32%	37%	17%
Anxiety	20%	37%	7%
**PSS** (perceived stress scale, 0–40): Median (1^st^ quartile, 3^rd^ quartile, semi-interquartile range)	17.0 (12.0, 24.0, 6.0)	18.0 (12.5, 21.5, 4.5)	7.5 (5.0, 17.0, 4.75)
***OAB symptom scores: (mean ± SD)***			
**ICIQ-UI** (urinary incontinence, 0–21):	12.0 ± 4.9	6.6 ± 5.7	1.4 ± 2.0
**UDI-6** (urogenital distress inventory, 0–24):	12.6 ± 5.6	11.0 ± 5.3	0.9 ± 1.4
**IIQ-7** (incontinence impact questionnaire, 0–28):	8.8 ± 8.2	4.9 ± 7.0	0.1 ± 0.4
**OAB-q** quality of life subscale (13–78):	29.7 ± 16.9	25.1 ± 14.9	2.0 ± 3.0
**OAB-q** symptom bother subscale (6–36):	19.1 ± 6.6	14.2 ± 7.4	2.2 ± 2.8
**ICIQ-OAB** (overactive bladder, 0–16):	9.3 ± 2.6	7.3 ± 4.1	2.0 ± 1.5
**USS** (urgency severity scale, 0–3):	2.1 ± 0.7	1.9 ± 0.8	0.5 ± 0.6
Numeric rating scale of **urgency** (0–10):	6.1 ± 2.6	5.9 ± 2.8	0.4 ± 0.6
Numeric rating scale of **frequency** (0–10):	6.4 ± 2.6	6.2 ± 2.3	0.6 ± 0.9

### Comparison of perceived stress levels between OAB, IC/BPS and controls

**Figure 1:**
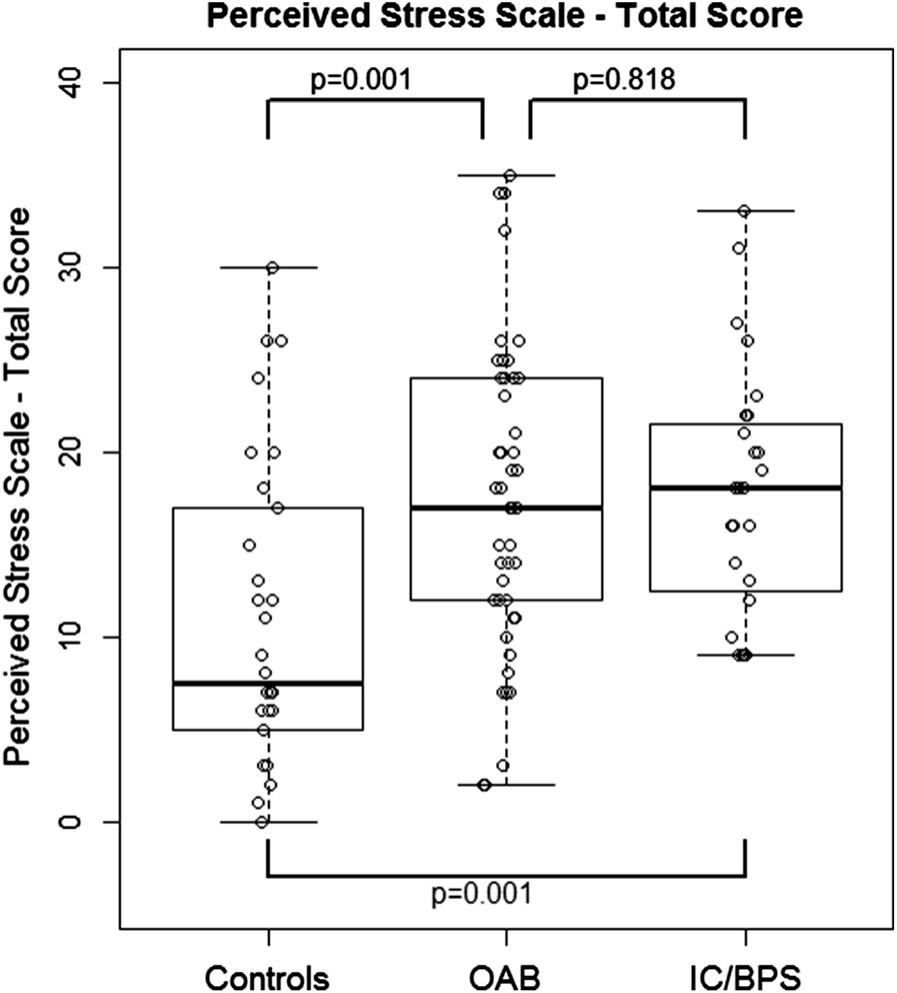
**Comparison of perceived stress levels among groups (Wilcoxon rank-sum tests).**

The total scores of the PSS are illustrated in Figure 1 and Table 1. On univariate analysis, OAB patients reported significantly higher psychological stress levels on the PSS compared to age-matched healthy controls (median 17.0, versus 7.5, p = 0.001, Wilcoxon sum rank test). There was no difference in psychological stress levels reported by OAB and IC/BPS patients (median 17.0 versus 18.0, p = 0.818). As expected, IC/BPS patients also reported significantly higher psychological stress levels on the PSS compared to controls (median 18.0 versus 7.5, p = 0.001). Because IC/BPS patients were more likely to be females and older than OAB patients, we incorporated age and sex into our multivariate modeling. On multivariate analysis, PSS remained significant different between OAB and controls (p = 0.001) after adjusting for age and sex.

### Correlation between perceived stress levels and urinary symptoms among OAB patients

Among OAB patients, high perceived stress levels on the PSS was positively correlated to total scores on ICIQ-UI (Spearman’s correlation coefficient r_s_ = 0.393, p = 0.007), UDI-6 (Spearman’s correlation coefficient = 0.314, p = 0.028), IIQ-7 (Spearman’s correlation coefficient = 0.393, p = 0.005), and quality of life subscale on the OAB-q (Spearman’s correlation coefficient = 0.326, p = 0.029), see Table 2. No significant correlation between perceived stress levels and the following instruments was observed: ICIQ-OAB, symptom bother subscale on the OAB-q, USS, and the numeric ratings of their urgency or frequency (0 to 10).

**Table 2:** **Correlation of perceived stress levels and urinary symptoms among OAB patients (Spearman’s correlation tests)**

	**Correlation analysis between PSS total scores and OAB symptoms:**	
	**Spearman’s correlation coefficient (r**_**s**_ **)**	**p-value**
**ICIQ-UI** (urinary incontinence, 0–21)	0.393	0.007*
**UDI-6** (urogenital distress inventory, 0–24)	0.314	0.028*
**IIQ-7** (incontinence impact questionnaire, 0–28)	0.393	0.005*
**OAB-q** quality of life subscale (13–78)	0.326	0.029*
**OAB-q** symptom bother subscale (6–36)	0.195	0.189
**ICIQ-OAB** (overactive bladder, 0–16)	0.205	0.158
**USS** (urgency severity scale, 0–3)	0.127	0.390
Numeric rating scale of **urgency** (0–10)	0.235	0.090
Numeric rating scale of **frequency** (0–10)	0.260	0.068
***Specific items on the ICIQ-UI questionnaire:***		
**ICIQ-UI:** “How often do you leak urine?” (0–5)	0.328	0.020*
**ICIQ-UI:** “How much urine do you think you leak?” (0–6)	0.334	0.018*
**ICIQ-UI:** “How much does leaking urine interfere with your daily life?” (0–10)	0.270	0.058
***Specific items on the ICIQ-OAB questionnaire:***		
**ICIQ-OAB:** “How many times do you urinate during the day” (0–6)	0.104	0.473
**ICIQ-OAB:** “During the night, how many times do you have to urinate during the night, on average?”	0.052	0.722
**ICIQ-OAB:** “Do you have to rush to the bathroom to urinate?”	0.238	0.096
**ICIQ-OAB:** “Does urine leak before you can get to the bathroom?”	0.297	0.036*

*statistical significance, p < 0.05.

When the specific items of the ICIQ-UI questionnaire was examined separately in an exploratory analysis, two of the items (“how often do you leak urine?”, “how much urine do you think you leak?”) was also positively correlated to perceived stress levels on the PSS (Spearman’s correlation coefficient = 0.328, 0.334, and p-value = 0.020, 0.018, respectively).

When the four items of the ICIQ-OAB questionnaire was examined separately in an exploratory analysis (“how many times do you urinate during the day?”, “during the night, how many times do you have to wake up to urinate, on average?”, “do you have to rush to the bathroom to urinate?”, “does urine leak before you can get to the bathroom?), response on the urinary incontinence question (item 4) was positively correlated to perceived stress levels on the PSS (Spearman’s correlation coefficient = 0.297, p-value = 0.036). On the other hand, responses on the frequency question (item 1), nocturia question (item 2) and urgency question (item 3) did not show any significant correlation to the PSS (Spearman’s correlation coefficient = 0.104, 0.059, 0.238, and p-value = 0.473, 0.689, 0.096, respectively).

### Comparison of perceived stress levels between OAB patients with urgency incontinence versus mixed incontinence

OAB patients were recruited if they had urinary urgency, with or without urgency incontinence, in accordance with the 2002 ICS definition of OAB [7]. Patients with concomitant stress incontinence (or mixed incontinence) were also eligible if they reported predominant urgency incontinence by history. Overall, 98% of OAB patients in our cohort reported incontinence symptoms on the ICIQ-UI. Among them, 45% reported urgency incontinence without stress incontinence, and 53% reported mixed incontinence (urgency and stress incontinence) on the ICIQ-UI. There was no difference in PSS between OAB patients with urgency incontinence versus those with mixed incontinence (urgency incontinence PSS = 15.7 ± 1.71, mixed incontinence PSS = 18.8 ± 1.5, p = 0.17). Because the percent of OAB patients without any incontinence was too small, we were not able to compare the PSS between OAB patients with incontinence versus OAB patients without incontinence.

### Influence of childhood traumatic history, anxiety, and depression on perceived stress levels

Stress levels may be influenced by psychosocial factors such as childhood traumatic events, anxiety and depression. These factors were assessed using the childhood traumatic event scale and the hospital anxiety and depression scale (HADS), respectively [16,17]. OAB patients with a childhood history of sexual trauma or physical trauma reported higher stress levels on the PSS compared to OAB patients without such a history (PSS of 20.6 ± 1.8 versus 15.2 ± 1.4, p = 0.048). OAB patients with higher anxiety scores (HADS-A ≥ 8) had higher stress levels than those with lower anxiety scores (HADS-A < 7), PSS of 23.1 ± 1.3 versus 11.9 ± 1.1, p < 0.0001. OAB patients with higher depression scores (HADS-D ≥ 8) had higher stress levels than those with lower depression scores (HADS-D < 7), PSS of 22.9 ± 1.2 versus 15.1 ± 1.4, p = 0.014.

## Discussion

There are two findings in this study: (1) OAB patients reported psychological stress levels that were as high as IC/BPS patients, and significantly higher than healthy controls, (2) among OAB patients, there were significant positive correlation between psychological stress levels perceived by patients and the severity of urinary incontinence symptoms (ICIQ-UI), and its impact on quality of life (UDI-6, IIQ-7, OAB-q quality of life subscale).

The clinical implication of our findings is that when treating patients with OAB, a psychological component should be evaluated as possibly contributing to the nature and severity of the lower urinary tract symptoms. Psychological stress might be a potential modifiable risk factor for urgency incontinence. In fact, a recent clinical study suggested that mindfulness-based stress reduction (MBSR) might reduce urgency incontinence episodes [19]. MBSR is a structured, group-based, mind-body intervention with the goal to teach participants stress reduction techniques by a variety of mediation practices, mindful yoga, and discussion of the relation between stress, illness and health. In a non-randomized study that recruited seven women, Baker et al. showed that MBSR reduced urgency incontinence episodes more than it reduced urinary frequency [19].

We have identified a potential link between psychological stress and UI symptoms. Since there were positive correlation between the level of stress and a number of measures (a “dose–response” gradient), this supported the possibility of a causal relationship instead of a pure coincidence. However, the directionality of the relationship cannot be ascertained in this case control study. While it is not surprising that incontinence can increase psychological stress (UI → stress), the reverse may also occur, i.e. high psychological stress may exacerbate existing UI symptoms (stress → UI). The interaction may also be bi-directional (stress ↔ UI). A prospective study is needed to further clarify the causality or directionality of the association between psychological stress and lower urinary symptoms.

The second scenario (stress → UI) is biologically plausible. In animal studies, exposure of female rats to repeated water avoidance stress caused an increase in micturition frequency, reduction of micturition volume, and bladder hypersensitivity [20,21]. Exposure of male rats to rotating stressors had similar effects [22]. Exposure of male rats to repeated social defeat stress had the opposite effect: it caused urinary retention and non-voiding bladder contractions [23]. These stress-induced bladder dysfunctions may be mediated by corticotropin releasing factor (CRF), which functions both as a hormone to regulate the activity of the hypothalamic-pituitary-adrenal (HPA) axis, and as a neurotransmitter in the central nervous system to modulate the function of the pontine micturition center and the descending micturition pathways [23,24]. Currently, it is unknown if there is an alteration in CRF response, HPA axis, or diurnal cortisol levels in patients with OAB. Other factors such as low-grade systemic inflammation or central nervous system changes may be involved [25]. Such changes have been observed in IC/BPS patients [26]. Since there is considerable overlap of urinary symptoms between OAB and IC/BPS (e.g. urgency, frequency) [4,8], these questions deserve further investigation in OAB.

It is not clear why a positive correlation was observed between stress levels and urinary incontinence symptoms, but not with frequency or urgency symptoms. Our results were in agreement with the study by Knight et al. which also found no differences in life stress scores between “OAB dry” patients and controls [5]. Unfortunately the authors did not recruit “OAB wet” patients (with incontinence) for comparison. With IC/BPS, there is a positive correlation between stress levels and the severity of urgency symptom [3]. This correlation becomes progressively stronger in patients with more severe IC/BPS symptoms. In contrast, with OAB we have not observed a correlation between psychological stress levels and the severity of urgency symptom. We did not observe a difference in stress levels between OAB patients with urgency incontinence versus mixed incontinence. However, the sample size might be too small to examine this issue.

The current study has limitations: (1) the case control design did not prove causation or directionality; (2) our cohort did not permit a comparison between “OAB wet” and “OAB dry” patients since 98% of our patients have urgency incontinence (with or without stress incontinence); and (3) the correlation analysis was based on subjective data reported by the patients instead of objective findings (e.g. pad weight, urodynamics). While having objective data might add value to the analysis, they cannot fully replace symptom data or quality of life data, since these patient-reported data reflected patients’ own experience of their conditions.

## Conclusions

OAB patients reported psychological stress levels that were as high as IC/BPS, and significantly higher than healthy controls. There was a positive correlation between perceived stress levels and urinary incontinence symptoms, and its impacts on quality of life among OAB patients.

### Availability of supporting data

Supporting data from the study will be provided to other researchers upon request.

## Abbreviations

HADS: Hospital anxiety and depression scale
IC/BPS: Interstitial cystitis/ bladder pain syndrome
ICIQ-OAB: International consultation on incontinence – overactive bladder
ICIQ-UI: International consultation on incontinence – urinary incontinence
IIQ-7: Incontinence impact questionnaire short form
OAB: Overactive bladder
PSS: Perceived stress scale
UDI-6: Urogenital distress inventory short form
USS: Urgency severity scale
